# Intrinsic superflat bands in general twisted bilayer systems

**DOI:** 10.1038/s41377-022-00838-0

**Published:** 2022-05-30

**Authors:** Hongfei Wang, Shaojie Ma, Shuang Zhang, Dangyuan Lei

**Affiliations:** 1 Department of Materials Science and Engineering, City University of Hong Kong, Kowloon, Hong Kong 999077 China; 2 Department of Physics, University of Hong Kong, Hong Kong, 999077 China; 3 Department of Electrical and Electronic Engineering, University of Hong Kong, Hong Kong, 999077 China

**Keywords:** Nanophotonics and plasmonics, Photonic crystals

## Abstract

Twisted bilayer systems with discrete magic angles, such as twisted bilayer graphene featuring moiré superlattices, provide a versatile platform for exploring novel physical properties. Here, we discover a class of superflat bands in general twisted bilayer systems beyond the low-energy physics of magic-angle twisted counterparts. By considering continuous lattice dislocation, we obtain intrinsic localized states, which are spectrally isolated at lowest and highest energies and spatially centered around the AA stacked region, governed by the macroscopic effective energy potential well. Such localized states exhibit negligible inter-cell coupling and support the formation of superflat bands in a wide and continuous parameter space, which can be mimicked using a twisted bilayer nanophotonic system. Our finding suggests that general twisted bilayer systems can realize continuously tunable superflat bands and the corresponding localized states for various photonic, phononic, and mechanical waves.

## Introduction

Twisted bilayer systems of two-dimensional (2D) materials, especially for graphene^1,2^ and transition metal dichalcogenides (TMDCs)^3,4^, have recently been employed to explore various physics and applications such as spin-polarized phases^5–8^ and unconventional superconductivity^9–11^. For general twist angles, the scale of moiré superlattices ranges in size from unit cells of 2D materials to infinity^12–14^. The structural flexibility further makes twisted van der Waals heterostructures a versatile and tunable platform^15–18^. However, these characteristic behaviors, such as Mott insulating states^19–22^ and superconducting states^6,23–25^, always occur at particular discrete twist angles between two sheets, denoted as magic angles^10,26–28^, which are sensitive to tiny perturbations in structural manipulation. At present, moiré flat bands and topological bands near the Fermi level underlying the above extraordinary progress have been fully studied both in theory and experiment^29–33^. However, these novel physics and phenomena require precise control of twist angles which are difficult to generalize to distinct artificial materials for various wave systems. General effects and exotic physical phenomena of twisted bilayer systems insensitive to twist angles remain out of reach.

In this work, we discover the robust presence of a class of superflat bands in general twisted bilayer systems proved by the tight-binding model (TBM) with negligible next-nearest-neighbor intralayer hoppings. Using the effective macroscopic potential well model (PWM) with spatially modulated couplings, we show that for small twists, localized states definitely appear centered on the AA stacked region (with deepest potential well) at isolated lowest and highest energies, manifesting *C*_6_ and *C*_3_ symmetries, respectively. Such localized states present negligible inter-cell coupling, forming superflat bands for general twisted bilayer systems, which is corroborated by exact TBM calculations. We further implement superflat bands and the corresponding localized states via twisted bilayer nanophotonic platforms. Importantly, these superflat bands arise for a continuous set of small angles and do not require fine tuning to the specific magic angles, being readily implementable for various wave systems and introducing an extremely large density of states (DOS) for lasing^34^, sensing^35^, and light-matter interactions^36^.

## Results

### Superflat bands and localized states

**Fig. 1:**
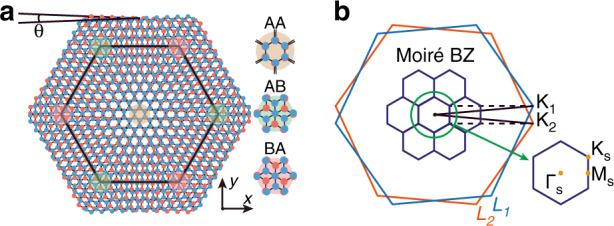
Lattices and Brillouin zones (BZs) of generic twisted bilayer systems. **a** Schematic of general twisted bilayer systems with the twist angle *θ*, where the largest black hexagon denotes moiré superlattices including AA and AB/BA stacked lattices. Blue (red) dots represent the sites in *L*_1_ (*L*_2_). **b** Schematic of BZs. Sky blue and red hexagons represent first BZs for *L*_1_ and *L*_2_ while black hexagons represent moiré BZs

General twisted bilayer systems display alternating patterns between AA and AB/BA stacked lattices (i.e., the A (B) site from the upper layer is perfectly aligned with the A/B (A) site from the lower layer), as illustrated in Fig. 1a. In momentum space, rotated unit cells in two layers cause a relative rotation (*θ*) of first Brillouin zones (BZs), generating an effective moiré BZ (see Fig. 1b). Periodic moiré superlattice has the lattice constant ${a}_{M}=\frac{a}{2{\rm{sin}}(\theta /2)}$, where *a* is the lattice constant of primitive unit cells (with a hexagonal p6m symmetry of wallpaper groups). We assume that the hopping rate between every two sites (*i* ≠ *j*) decays exponentially as a function of distance ∣***r***_*ij*_∣, i.e., ${t}_{ij} \sim {A}_{0}{e}^{-\gamma | {{{{\boldsymbol{r}}}}}_{ij}| }$, because the classical electronic and photonic systems always allow the overlap of exponential-type wave functions^37–40^. Here *γ* represents the decay rate and *A*_0_ is the normalized coefficient constraining the energy scale. In addition, negligible next-nearest-neighbor hoppings of intralayer sites restrict the range of *t*_*ij*_ in the following form

1 $${A}_{0}{e}^{-\gamma a/\sqrt{3}}={t}_{0},{A}_{0}{e}^{-\gamma a}\to 0$$

**Fig. 2:**
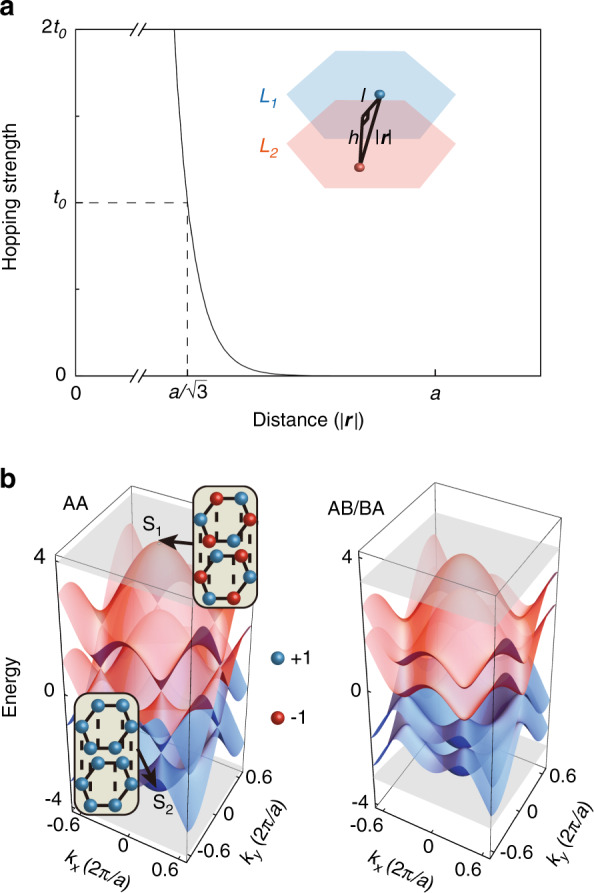
Generic hopping function and band structures of AA and AB/BA stacked lattices. **a** Hopping strength function that decays exponentially with the independent variable of site-to-site distance ***r***, where only the nearest-neighbor hoppings for intralayer sites are considered. **b** Rigorous band structures of AA and AB/BA stacked lattices calculated by the TBM with $h=a/\sqrt{3}$. The insets show the field distributions of S_1_ (highest energy) and S_2_ (lowest energy) located at Γ point, implying *C*_3_ and *C*_6_ symmetries, respectively

Without losing generality, we set the unit hopping *t*_0_ = 1 in the following analysis. To ensure the dominance of nearest-neighbor hoppings accurately, we further choose *γa* ~ 30 corresponding to *t*_*ij*_(*a*) ~ 10^−5^ ≪ *t*_0_. An exact hopping strength curve is displayed in Fig. 2a, where the spatial distance $| {{{{\boldsymbol{r}}}}}_{ij}| =\sqrt{{l}^{2}+\rho {h}^{2}}$, *l* and *h* represent the intralayer and interlayer distances, respectively. *ρ* = 0 (*ρ* = 1) stands for *i* and *j* located at the same (distinct) layers. We model general spinless twisted bilayer systems with the TB Hamiltonian

2 $${H}_{{{{\rm{TB}}}}}=-\mathop{\sum}\limits_{\langle i,j\rangle }{t}_{ij}^{\rho = 0}{c}_{i}^{{\dagger} }{c}_{j}-\mathop{\sum}\limits_{i,j}{t}_{ij}^{\rho = 1}{c}_{i}^{{\dagger} }{c}_{j}+\mathop{\sum}\limits_{i}\epsilon {c}_{i}^{{\dagger} }{c}_{i}$$

where ${c}_{i}^{({\dagger} )}$ corresponds to the creation (annihilation) operator at the site *i*, and *ϵ* is the inherent potential which is considered as zero in general systems. This allows us to perform the exact analysis for moiré superlattices and provide numerical support for the following detailed models.

Furthermore, we calculate the AA and AB/BA stacked band structures under the above hopping relation using the analytical TBM^41,42^. The lowest/highest energies of first BZs (located at Γ point, i.e., the center of blue and red hexagons in Fig. 1b) can be reduced to

3 $$\begin{array}{l}\;\;\;\;\;{E}_{{{\Gamma }}}^{{{{\rm{AA}}}}}=\pm \!\! ({t}_{ij}(h)+3{t}_{0}),\\ {E}_{{{\Gamma }}}^{{{{\rm{AB/BA}}}}}=\pm \!\! \dfrac{1}{2}({t}_{ij}(h)+\sqrt{{{t}_{ij}(h)}^{2}+36{t}_{0}^{2}})\end{array}$$

Algebraic derivation reveals that for highest (or lowest) bands AA stacked lattices always have higher (or lower) energies than AB/BA stacked lattices, forming a natural potential difference, i.e., $| {E}_{{{\Gamma }}}^{{{{\rm{AA}}}}}| \,>\, | {E}_{{{\Gamma }}}^{{{{\rm{AB/BA}}}}}|$, unless *h* → +*∞*, that is, $| {E}_{{{\Gamma }}}^{{{{\rm{AA}}}}}| =| {E}_{{{\Gamma }}}^{{{{\rm{AB/BA}}}}}|$. Such a relevant energy difference provides a spatial potential well where the deeper potential is located at the AA stacked region with effective masses m* ~ ±2*ℏ*^2^/*t*_0_. These effective masses remain almost constant at arbitrary locations within the moiré superlattices, with the details given in Supplementary Note 1. Here we show a specific case with $h=a/\sqrt{3}$ (see Fig. 2b), where band structures of AA and AB/BA stacked lattices match well with our analysis. Two states (S_1_ and S_2_) with highest/lowest energies at Γ point of AA stacked lattices present *C*_3_ and *C*_6_ symmetries, respectively, preserved by irreducible representations in the orthogonal eigenspace, which are the crucial prerequisite for forming superflat bands as following discussions.

**Fig. 3:**
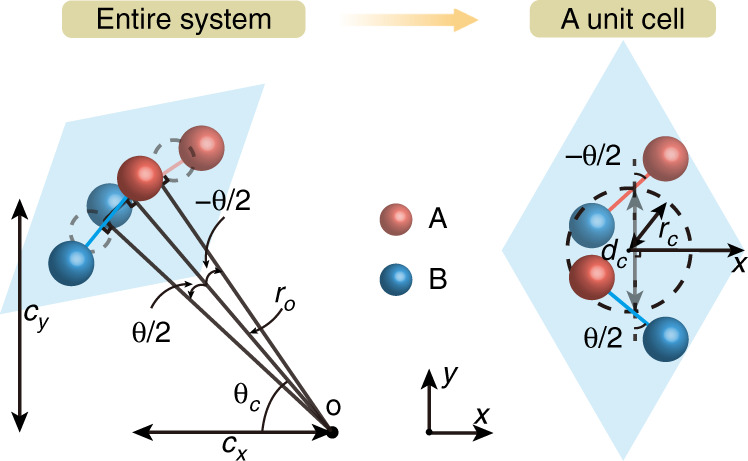
Lattice dislocation mapping. Schematic of the lattice dislocation under specific *r*_*o*_, *θ*, and *θ*_*c*_ (left panel), which can be characterized in terms of *θ*_*c*_, *r*_*c*_, and *d*_*c*_ (right panel)

**Fig. 4:**
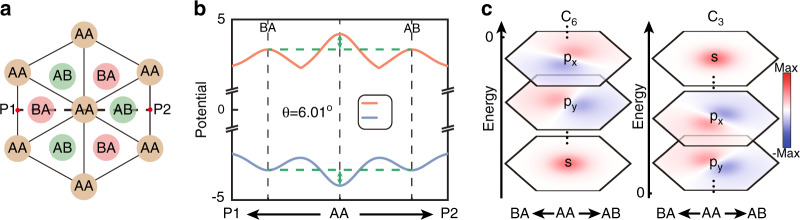
Localized states obtained by the macroscopic effective energy potential well method. **a** Geometry arrangements of alternating AA and AB/BA stacked lattices and a typical dotted line P1P2 used for the analysis in (**b**). **b** Two potentials of *V* vary with the spatial parameter between P1 and P2 with *θ* = 6.01^∘^ and $h=a/\sqrt{3}$, which are obtained by the energies of S_1_ and S_2_. Such a description is certainly valid for six different *θ*_*c*_. **c** Calculated eigenstates arranged from the lowest to highest energy. Panels are labeled as *s* and *p*_*x*,*y*_ states for both *E* < 0 and *E* > 0 cases. Such filed distributions reveal the corresponding *C*_6_ and *C*_3_ symmetries consistent with the exact eigenstates at Γ point of band structures of AA stacked lattices

In the vicinity of lowest/highest energies of AA stacked lattices, the previous low-energy theory describing moiré bands is invalid^29^. A concise physical picture can be constructed to depict this system as illustrated in Fig. 3. The distorted lattices along the azimuth *θ*_*c*_ = *nπ*/3, *n* = 1, 2, . . . , 6, centered around AA stacked lattices, reflect essential characteristics of the potential well. Specifically, for the distorted lattice with a distance from the center of AA stacked region *r*_*o*_, the coordinates of lattice center are (*c*_*x*_, *c*_*y*_) = *r*_*o*_(cos(*θ*_*c*_), sin(*θ*_*c*_)). The geometric center of A and B sites is shifted and projected on a specific circle with radius *r*_*c*_ = 2*r*_*o*_sin(*θ*/4). The distance in x–y plane from one center to another center for two layers is *d*_*c*_ = 2*r*_*o*_sin(*θ*/2). Here, *r*_*c*_ and *d*_*c*_ are independent of *θ*_*c*_. In the vicinity of AA stacked region, dislocated lattices for any *r*_*o*_ and *θ*_*c*_ allow for modeling on a scale of unit cells. A typical case for *n* = 0 is displayed in Fig. 3 (right panel). The Hamiltonian around Γ point characterizing lattice distortions of the system, ${{\Phi }}=\{{\phi }_{A}^{1},{\phi }_{B}^{1},{\phi }_{A}^{2},{\phi }_{B}^{2}\}$, takes the form^43^

4 $$H({{{\boldsymbol{k}}}})=\left(\begin{array}{cc}{h}_{1}&F\\ {F}^{T}&{h}_{2}\end{array}\right)$$

where ${h}_{1,2}=-{\sigma }_{x}\mathop{\sum }\nolimits_{i = 0}^{2}{t}_{i}+{\sigma }_{y}(\pm a\frac{{t}_{1}-{t}_{2}}{2}{k}_{x}+\sqrt{3}a\frac{{t}_{1}+{t}_{2}}{2}{k}_{y})$ and the wavevector ***k*** = {*k*_*x*_, *k*_*y*_}. *σ*_*x*,*y*_ are the Pauli matrices acting in sublattice space of single layers. *t*_1_ and *t*_2_ correspond to inter-cell hoppings between A and B sites for single layers along two distinct basis vectors, respectively, which are equal for zero *θ* or unequal (and exchanged in another layer) for nonzero *θ*. Besides, the exact derivation manifests that *t*_1_ (*t*_2_) only grows as *θ* decreases (increases) (Supplementary Note 2). The off-diagonal function *F* = {*f*_11_, *f*_12_; *f*_21_, *f*_22_} represents the spatially modulated interlayer hoppings, which can be obtained analytically according to Fig. 3 and single depends on *r*_*o*_ under a given *θ*, as described in Supplementary Note 2. By diagonalizing Eq. (4), that is, *E*(Γ) = *PH*(Γ)*P*^−1^ (*P* is an invertible matrix), the spatial potential *V*(*r*_*o*_) is given by the function $\{{\rm{min}}\left(E\right.({{\Gamma }}),{\rm{max}}\left(\right.E({{\Gamma }})\}$, which is related to the energies of S_1_ and S_2_ in distorted lattices. In Figs. 4a, b, we show a specific cross section P1P2 (with length $\sqrt{3}{a}_{M}$) for *θ*_*c*_ = 0 or *π*, where *θ* = 6.01^∘^ and $h=a/\sqrt{3}$. The negative m* matches with S_1_ and has positive potential energies, while the positive m* matches with S_2_ and has negative potential energies. One sees that potential exhibits local valley (peak) characteristic for positive (negative) m*. The potential difference between AA and AB/BA stacked lattices always holds making the central AA stacked lattice become the global extrema of potential, which supports a 2D potential well of finite depth.

Consider the isotropy distortion approximation in the vicinity of central AA stacked region. The system can be regarded as the evolution of a spinless particle with effective mass m* in a given *V*(*r*_*o*_) potential well. We describe this process using the time-independent Schrödinger-like equation with eigenstates Ψ, given by

5 $$[-{\hslash }^{2}/2{m}^{* }({\partial }_{x}^{2}+{\partial }_{y}^{2})+V({r}_{o})]{{\Psi }}=E{{\Psi }}$$

The solutions of Eq. (5) are shown in Fig. 4c. Discrete energy levels correspond to different orders of Ψ manifesting the arrangement of *s*, *p*_*x*,*y*_, ..., *p*_*x*,*y*_, *s* states from lowest to highest energies. The first half of these states (*E* < 0) is composed of S_2_ with *C*_6_ symmetry, while the second half (*E* > 0) is composed of S_1_ with *C*_3_ symmetry. At lowest and highest energies, *s* states isolated from the continuous bulk energy spectrum exhibit ideal confinement, which can be understood from the confining *V*(*r*_*o*_) induced by intrinsic spatial hopping modulations.

**Fig. 5:**
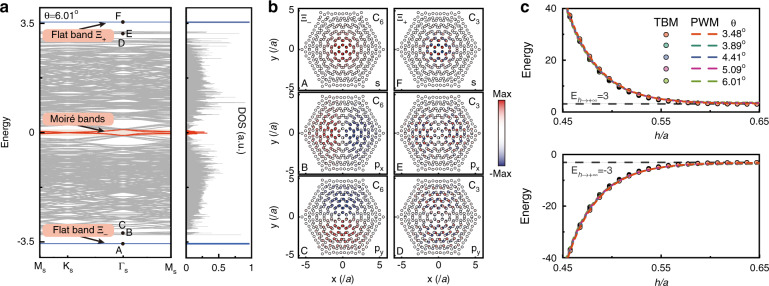
Superflat bands and corresponding localized states obtained by the tight-binding model method. **a** Band structures and related DOS obtained by the TBM with *θ* = 6.01^∘^ and $h=a/\sqrt{3}$. Two superflat bands (Ξ_−_ and Ξ_+_) are labeled in blue located at lowest and highest energies with extremely sharp DOS. Besides, typical moiré bands labeled in red appear around the zero energy with divergent DOS. **b** Various eigenstates for Ξ_−_ and Ξ_+_ bands and adjacent bands, which are arranged from the lowest to highest energy, i.e., A–F, corresponding to *s*, *p*_*x*,*y*_, ..., *p*_*x*,*y*_ and *s* states. **c** The energies of superflat bands that vary with *h* around $a/\sqrt{3}$. Different cases of *θ* (i.e., 3.48^∘^, 3.89^∘^, 4.41^∘^, 5.09^∘^, and 6.01^∘^) have also been represented in different colors. Faint circles and dark dotted lines correspond to the results in TBM and PWM, respectively, while faint gray dotted lines indicate the bulk energies of AA stacked lattices for infinite *h*

To further demonstrate the properties of general periodic twisted bilayer systems, we calculate band structures of moiré superlattices using the TBM with the hopping function of Fig. 2a. A representative result for *θ* = 6.01^∘^ and $h=a/\sqrt{3}$ is plotted in Fig. 5a. Four subbands (red curves) near the zero energy for spinless particles are fully consistent with typical moiré bands, corresponding to the divergent DOS, see the right panel of Fig. 5a. Whereas for the lowest and highest energies, superflat bands (blue curves) emerge in isolation accompanied by extremely large DOS, labeled as Ξ_−_ and Ξ_+_. Figure 5b shows typical eigenstates at Γ_S_ point of Ξ_−_, Ξ_+_ and their adjacent bands. Ξ_−_ (A) and Ξ_+_ (F) correspond to *s* states formed by S_2_ and S_1_, respectively. The eigenstates for *E* < 0 (A–C) and *E* > 0 (D–F) cases are consistent with the solution of the above continuous PWM in Fig. 4c. We further study the energies of Ξ_−_ and Ξ_+_ with different *h* and *θ* both in TBM and PWM, as displayed in Fig. 5c. Since such superflat bands are constrained by the potential of AA stacked lattices, i.e., ${E}_{{{\Gamma }}}^{{{{\rm{AA}}}}}$, the energies of Ξ_−_ and Ξ_+_ vary exponentially with *h* in a wide range of *θ*. As *h* → +*∞*, the energies of Ξ_−_ and Ξ_+_ tend to −3*t*_0_ and 3*t*_0_, respectively, merging into the bulk energy spectrum progressively (Supplementary Note 3).

### Nanophotonic implementation

**Fig. 6:**
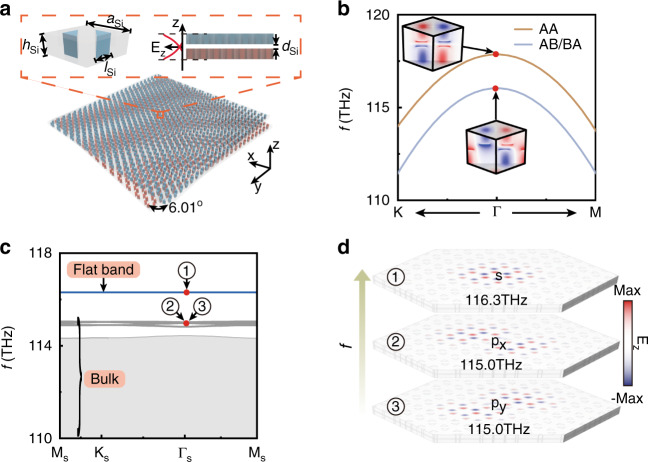
Nanophotonic design and results for the superflat band and localized state. **a** Schematic of twisted bilayer PCs made of silicon and air materials, with graphene-like lattices in each slab. The left inset displays the three-dimensional unit cell structure of single layers. The right inset presents the cross section of twisted bilayer PCs and the amplitude (E_z_) of fundamental modes along the z direction. **b** Band structures of AA and AB/BA stacked PCs near Γ point. The eigenfrequencies for AA stacked PCs are significantly greater than that of AB/BA stacked PCs under the same essential parameters (i.e., *a*_Si_, *h*_Si_, *l*_Si_, and *d*_Si_). The insets represent the eigenstates with *C*_3_ symmetry at Γ point. **c** Band structures of moiré superlattices with twist angle 6.01^∘^. The superflat band (blue) is separated from the rest of bands. **d** Typical eigenstates (E_z_) of moiré superlattices at Γ_*S*_ point of moiré BZs on the superflat band and adjacent bands, i.e., *s* and *p*_*x*,*y*_ states. *s* state exhibits well-confined features with *C*_3_ symmetries, leading to intrinsic superflat bands. Nonflat bands composed of *p*_*x*,*y*_ states are enumerated as the comparison. Eigenfrequencies and the corresponding electromagnetic fields are solved by COMSOL

To realize superflat bands and the corresponding localized states in nanophotonic systems, we propose a twisted bilayer photonic crystal (PC) composed of an air layer and two twisted PC slabs, as shown in Fig. 6a. Single PC slab has a *C*_6*v*_ lattice with lattice constant *a*_Si_ = 1.5 μm filled with air, where the sublattices are composed of silicon triangular prisms (refractive index *n*_Si_ = 3.46) with sidelength *l*_Si_ = 0.35*a*_Si_ and height *h*_Si_ = 0.5*a*_Si_ (see the left inset of Fig. 6a). The air layer with a thickness of *d*_Si_ = 0.2*a*_Si_ is sandwiched between two twisted PC slabs (see the right inset of Fig. 6a). The entire structure is embedded in perfect metal in the stacking direction forming a conservative system (here the transverse magnetic (TM) polarization is considered). We also provide the design under open systems as support (Supplementary Note 4).

Owing to the long-wavelength limit of dielectric PCs, the lowest band with linear dispersion near Γ point exhibits a fixed lowest frequency 0 leading to the absence of superflat bands with *C*_6_ symmetric states (Supplementary Note 5)^44^. So we only present the case possessing *C*_3_ symmetric states. Figure 6b shows band structures near Γ point for AA and AB/BA stacked PCs with given parameters in Fig. 6a. Because the electromagnetic fields are concentrated in carefully designed triangular prisms, this system can match well with TBMs^44^. The *C*_3_ symmetric eigenstates of these two bands preserve particular frequency difference ensuring that the states located in AA stacked lattices is isolated from bulk spectra of twisted bilayer PCs (see the insets of Fig. 6b). Then, we calculate the band structure of twisted bilayer PCs with twist angle 6.01^∘^, as plotted in Fig. 6c. The superflat band (blue) is observed at the frequency 116.3 THz, describing well-confined *s* states with *C*_3_ symmetry, as shown in the top panel of Fig. 6d. Adjacent bands exhibit multipole states of moiŕe superlattices accompanied by worse localization capabilities. For example, *p*_*x*,*y*_ states form crossed and nonflat bands, see Fig. 6c and the middle and bottom panels of Fig. 6d.

Note that such a design process exactly focuses on a single mode of the triangular prism (e.g., the fundamental mode above, which is therefore located in several lower bands). Despite the robustness of localized states, the interaction of different order modes of the triangular prism may merge the superflat bands into upper adjacent bands, which should be avoided when setting essential parameters of the system (Supplementary Note 6).

## Discussion

The intrinsic superflat bands in our work have the property of isolated energy spectra without mode hybridization between different bands, so that the corresponding eigenstates have a clear and highly symmetrical phase distribution, as shown in Figs. 4c and 5c. The localized eigenstates are almost insensitive to periodic moiré superlattice boundaries, which is understood as the origin of superflat bands and can be described by the PWM. The carried *C*_3_ and *C*_6_ symmetries distinguished from moiré flat bands formed by the four-band reconstruction (moiré bands) near the zero energy have not been fully discussed before^9–11,26^. Recently, we notice that a displacement electric field is applied in specific twisted bilayer systems (e.g., graphene and boron nitride heterostructure) to study the valley topology of moiré bands^45,46^. In our system, this is equivalent to yielding a nonzero ∣*ϵ*∣ with distinct signs for two layers. The energies of superflat bands will be corrected corresponding to a shift *g*(∣*ϵ*∣), where *g*(∣*ϵ*∣) ≥ 0 and grows as ∣*ϵ*∣ increases, see the details given in Supplementary Note 7. Apart from that, nonzero ∣*ϵ*∣ cannot affect the presence of superflat bands and localized states.

In conclusion, combining theoretical PWM analysis and TBM calculation, we have demonstrated a class of superflat bands with *C*_6_ and *C*_3_ symmetric states for small twists in general twisted bilayer systems. The dislocated lattices formed by the systematic hopping modulation create macroscopic effective potential wells centered around the AA stacked region, leading to the well-confined states described by the PWM. We also mimic these two effects in nanophotonic systems displaying the unique electromagnetic wave confinement. Notably, superflat bands and the corresponding localized states can be realized for continuous twist angles (distinct from the discrete set of twist angles in magic-angle physics), showing a class of generalized effects of twisted bilayer systems distinguished from the fragile topology. The concept of generalized localized states may inspire a shortcut technology for generating zero-dimensional localization, avoiding complex boundary splicing of (higher-order) topological insulators, which will greatly benefit the wave trapping and manipulation. The frequencies of superflat bands and the configurations of localized states can be adjusted by twist angles, and this offers an advanced platform for reconfigurable devices. Our results can be extended to photonics^47–49^, phononics, and mechanical waves, where ideal transport can be realized for integrated chips in information technologies.

## Methods

### Nanophotonic simulation

Numerical simulations for nanophotonic systems in this work are all performed using the 3D electromagnetic module of commercial finite-element simulation software (COMSOL MULTIPHYSICS). In solving the eigenvalues and eigenstates of AA, AB/BA, and moiré lattices in our silicon-air platform, the calculation regions are selected as hexagonal unit cells with side lengths $\frac{a}{\sqrt{3}}$, $\frac{a}{\sqrt{3}}$, and $\frac{a}{2\sqrt{3}{\rm{sin}}(\theta /2)}$, respectively, with *a* being 1.5um and *θ* being 6.01^∘^. Bottom and top boundaries along the stacking direction are set as perfect electric conductors. So only the transverse magnetic (TM) polarization is considered for the data in Fig. 6b and c, i.e., E_z_. Whereas 2D periodic directions satisfy Bloch’s theorem E_z_(***r*** + ***R***) = *e*^*i****k***⋅***R***^E_z_(***r***), where ***R*** is a real space lattice vector.

## Supplementary information

Supplementary Information for Intrinsic Superflat Bands in General Twisted Bilayer Systems

## Data Availability

The data that support the plots within this work and other related findings are available from the corresponding authors upon reasonable request.

## Code availability

Numerical simulations in this work are all performed using the 3D electromagnetic module of a commercial finite-element simulation software (COMSOL MULTIPHYSICS). All related codes can be built with the instructions in the “Methods” section.
