# Supplementary material for: Intrinsic superflat bands in general twisted bilayer systems
Source: Light Sci Appl. 2022 May 30;11:159. doi: 10.1038/s41377-022-00838-0 (PMC9148907; doi:10.1038/s41377-022-00838-0)
Supplement: Supplementary file 1 — Supplementary Information for Intrinsic Superflat Bands in General Twisted Bilayer Systems [file 41377_2022_838_MOESM1_ESM.docx]

**Supplementary Information for “****Intrinsic Superflat Bands in General Twisted Bilayer Systems”**

Hongfei Wang,^1, †^ Shaojie Ma,^2, †^ Shuang Zhang,^2,3,*^ and Dangyuan Lei^1,*^

^*^Corresponding authors: S. Zhang: shuzhang@hku.hk; D. Lei: dangylei@cityu.edu.hk

^1^*Department of Materials Science and Engineering, City University of Hong Kong, Kowloon, Hong Kong 999077, China*

^2^*Department of Physics, University of Hong Kong, Hong Kong 999077, China*

**Supplementary Note 1: Band structures of AA and AB/BA stacked lattices**

General twisted bilayer systems exist periodic arranged AA and AB/BA stacked lattices. For the interlayer distance $h$, the Hamiltonian of ideal AA stacked lattices with the hopping functions $t_{ij}(|\boldsymbol{r}_{ij}|)=A_{0}e^{-\gamma|\boldsymbol{r}_{ij}|}$ can be written as:

$H_{AA}=-h_{AA}\oplus h_{AA}-\tau_{x}\otimes\sigma_{0}t_{ij}\left( h \right),$ (1)

where

$h_{AA}=\left( \begin{matrix} 0 & t_{0}+t_{0}e^{i\boldsymbol{k}\cdot\boldsymbol{a}_{1}}+t_{0}e^{-i\boldsymbol{k}\cdot\boldsymbol{a}_{2}} \\ t_{0}+t_{0}e^{-i\boldsymbol{k}\cdot\boldsymbol{a}_{1}}+t_{0}e^{i\boldsymbol{k}\cdot\boldsymbol{a}_{2}} & 0 \end{matrix} \right),$ (2)

where $\tau_{x}$ acts as interlayer degree of freedom and $\sigma_{0}$ acts as sublattice degree of freedom. $\boldsymbol{k}=\{k_{x},k_{y}\}$ represents the momentum space. The highest and lowest energies for entire band structures appear at the $\Gamma$ point and read

${max: E}_{\Gamma}^{AA}=t_{ij}\left( h \right)+3t_{0}$, (3)

$min: E_{\Gamma}^{AA}=-(t_{ij}\left( h \right)+3t_{0})$. (4)

The Hamiltonian of ideal AB/BA stacked lattices can be written as

$H_{AB/BA}=-h_{AB/BA}^{1}\oplus h_{AB/BA}^{2}-\frac{1}{2}\tau_{x}\otimes\left( \sigma_{0}-\sigma_{z} \right)t_{ij}\left( h \right),$ (5)

where

$h_{AB/BA}^{1}=\left( \begin{matrix} 0 & t_{0}+t_{0}e^{-i\boldsymbol{k}\cdot\boldsymbol{a}_{0}}+t_{0}e^{-i\boldsymbol{k}\cdot\boldsymbol{a}_{2}} \\ t_{0}+t_{0}e^{i\boldsymbol{k}\cdot\boldsymbol{a}_{0}}+t_{0}e^{i\boldsymbol{k}\cdot\boldsymbol{a}_{2}} & 0 \end{matrix} \right),$ (6)

$h_{AB/BA}^{2}=\left( \begin{matrix} 0 & t_{0}+t_{0}e^{i\boldsymbol{k}\cdot\boldsymbol{a}_{1}}+t_{0}e^{-i\boldsymbol{k}\cdot\boldsymbol{a}_{2}} \\ t_{0}+t_{0}e^{-i\boldsymbol{k}\cdot\boldsymbol{a}_{1}}+t_{0}e^{i\boldsymbol{k}\cdot\boldsymbol{a}_{2}} & 0 \end{matrix} \right),$ (7)

where the highest and lowest energies for entire band structures appear at the $\Gamma$ point and read

$max: E_{\Gamma}^{AB/BA}={\frac{1}{2}t}_{ij}\left( h \right)+\frac{1}{2}\sqrt{{t_{ij}\left( h \right)}^{2}+36t_{0}^{2}},$ (8)

$min: E_{\Gamma}^{AB/BA}=-\left( {\frac{1}{2}t}_{ij}\left( h \right)+\frac{1}{2}\sqrt{{t_{ij}\left( h \right)}^{2}+36t_{0}^{2}} \right).$ (9)

Notice that $\boldsymbol{a}_{0}=a\{1,0\},$ $\boldsymbol{a}_{1}=a\left\{ \frac{1}{2},\frac{\sqrt{3}}{2} \right\},$ $\boldsymbol{a}_{2}=a\left\{ \frac{1}{2},-\frac{\sqrt{3}}{2} \right\}$. Simple algebraic derivation shows that $|E_{\Gamma}^{AA}|>|E_{\Gamma}^{AB/BA}|$ always holds unless $h\to+\infty$ and $\left| E_{\Gamma}^{AA} \right|=|E_{\Gamma}^{AB/BA}|$.

Such an energy difference provides a prerequisite for the macroscopic effective potential wells and isolated localized states. Specifically, the energy of AA stacked regions in twisted bilayer systems near $\Gamma$ point can be clearly isolated from AB/BA stacked regions, which represents in such energy difference, the fields are only allowed to be distributed centered around the AA stacked area. The superflat bands in this range should also be isolated from the bulk energies unless $h$ becomes large enough or ideal AA and AB/BA stacked lattices are broken for large enough $\theta$.

According to the free electron gas model, the energy of bands for a quasi-free particle can be expanded as

$E_{\Gamma}=E_{\Gamma}^{0}+\frac{\boldsymbol{k}^{2}}{2}\frac{\partial^{2}}{\partial\boldsymbol{k}^{2}}E\left( \boldsymbol{k} \right),$ (10)

where $\frac{1}{2}\frac{\partial^{2}}{\partial\boldsymbol{k}^{2}}E\left( \boldsymbol{k} \right)=\frac{\hbar^{2}}{2m^{*}}$. For AA stacked lattices, $\frac{\partial^{2}}{\partial\boldsymbol{k}^{2}}E\left( \boldsymbol{k} \right)\sim\pm\frac{1}{2}t_{0}$, while for AB/BA stacked lattices, $\frac{\partial^{2}}{\partial\boldsymbol{k}^{2}}E\left( \boldsymbol{k} \right)\sim\pm\frac{3t_{0}^{2}}{\sqrt{t^{2}\left( h \right)+36t_{0}^{2}}}\to\pm\frac{1}{2}t_{0}$. So that the effective mass can be expressed as $m^{*}=\pm\frac{2\hbar^{2}}{t_{0}}$.

**Supplementary Note 2: The Hamiltonian induced by the** **interlayer dislocation**

In an approximation of continuous evolving, the Hamiltonian $H$ of distorted lattices in twisted bilayer systems can be derived from the Hamiltonian of graphene-type lattices^1,2^. For the twist angle $\theta=0$, with reference to the center of prismatic unit cells, coordinates of $a_{1}(a_{2})$ and $b_{1}(b_{2})$ sites correspond to $\boldsymbol{P}_{a_{1}(a_{2})}=(0,\frac{a}{2\sqrt{3}},\pm h/2)$ and $\boldsymbol{P}_{b_{1}(b_{2})}=(0,-\frac{a}{2\sqrt{3}},\pm h/2)$, where $h$ is the interlayer spacing. When $\theta\neq0$, the unit cell with the limited distance $r_{o}$ from the center of the system is equivalent to the offset trajectory ($r_{c}=2r_{o}sin(\theta/4)$) of center of two sites in $L_{1}/L_{2}$ layers as shown in Fig. S1. Here we focus on the evolution between AA and AB/BA stacks. So for a unit cell in these certain azimuth $\theta_{c}=\frac{n\pi}{3}$, $n=1,\ldots,6$, the general coordinate transformation of sublattice should be

$\boldsymbol{P}_{a_{1}(a_{2})}^{'}=R_{\pm\frac{\theta}{2}}\left( \boldsymbol{P}_{a_{1}(a_{2})} \right)+R_{\theta_{c}\mp\mathrm{asin} \left( \frac{d_{c}}{2r_{c}} \right)}([r_{c},0])$, (11)

$\boldsymbol{P}_{b_{1}(b_{2})}^{'}=R_{\pm\frac{\theta}{2}}\left( \boldsymbol{P}_{b_{1}(b_{2})} \right)+R_{\theta_{c}\mp\mathrm{asin} \left( \frac{d_{c}}{2r_{c}} \right)}([r_{c},0])$, (12)

where $d_{c}=2r_{o}sin(\theta/2)$ and $R_{\theta}$ represents the rotation operator that acts on the coordinate of sublattice. The tight-binding Hamiltonian in such gradient system can be written as

$H_{tb}=-t_{0}\sum_{\begin{aligned} \left\langle i,j \right\rangle\\ l \end{aligned}} a_{l,i}^{\dagger}b_{l,j}-t\left( \left| \boldsymbol{P}_{a_{1}}^{'}-\boldsymbol{P}_{a_{2}}^{'} \right| \right)\sum_{i} a_{1,i}^{\dagger}a_{2,i}-t\left( \left| \boldsymbol{P}_{b_{1}}^{'}-\boldsymbol{P}_{b_{2}}^{'} \right| \right)\sum_{i} b_{1,i}^{\dagger}b_{2,i}$ (13)

$-\sum_{i} \left( t\left( \left| \boldsymbol{P}_{a_{1}}^{'}-\boldsymbol{P}_{b_{2}}^{'} \right| \right)a_{1,i}^{\dagger}b_{2,i}+t\left( \left| \boldsymbol{P}_{a_{2}}^{'}-\boldsymbol{P}_{b_{1}}^{'} \right| \right)a_{2,i}^{\dagger}b_{1,i} \right)+H.c.,$


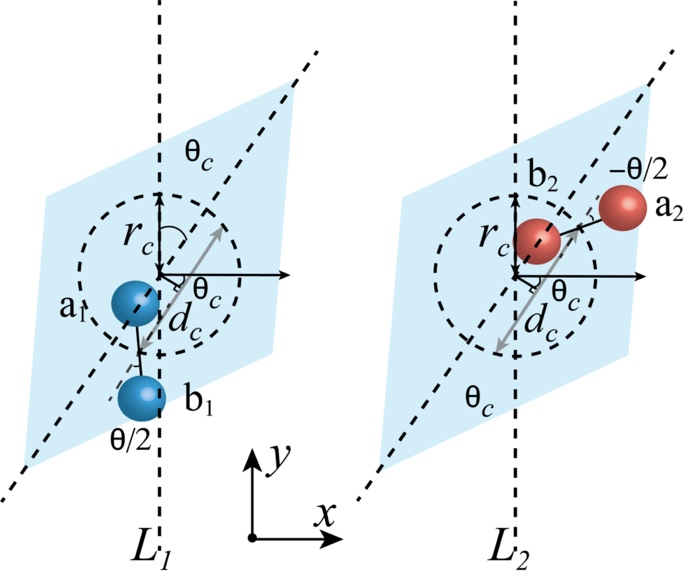


FIG. S1. Arrangement of twisted sites at different layers.

where $a_{l,i} (b_{l,i})$ represents the annihilation and creation operators on the sublattice $A (B)$ in layers $l=1,2$. Using the Fourier transform, the Bloch Hamiltonian can be further expressed as

$H_{tb}\left( \boldsymbol{k} \right)=\left( \begin{matrix} h_{1} & F \\ F^{T} & h_{2} \end{matrix} \right),$ (14)

and

$h_{1}=-\left( \begin{matrix} 0 & t_{0}+\sum_{j=1,2} t\left( \left| \boldsymbol{P}_{b_{1}}^{'}-\boldsymbol{P}_{a_{1}}^{'}-\left( -1 \right)^{j}\boldsymbol{c}_{j}^{1} \right| \right)e^{\left( -1 \right)^{j-1}i\boldsymbol{k}\boldsymbol{c}_{j}^{1}} \\ t_{0}+\sum_{j=1,2} t\left( \left| \boldsymbol{P}_{a_{1}}^{'}-\boldsymbol{P}_{b_{1}}^{'}-\left( -1 \right)^{j-1}\boldsymbol{c}_{j}^{1} \right| \right)e^{\left( -1 \right)^{j}i\boldsymbol{k}\boldsymbol{c}_{j}^{1}} & 0 \end{matrix} \right),$(15)

$h_{2}=-\left( \begin{matrix} 0 & t_{0}+\sum_{j=1,2} t\left( \left| \boldsymbol{P}_{b_{2}}^{'}-\boldsymbol{P}_{a_{2}}^{'}-\left( -1 \right)^{j}\boldsymbol{c}_{j}^{2} \right| \right)e^{\left( -1 \right)^{j-1}i\boldsymbol{k}\boldsymbol{c}_{j}^{2}} \\ t_{0}+\sum_{j=1,2} t\left( \left| \boldsymbol{P}_{a_{2}}^{'}-\boldsymbol{P}_{b_{2}}^{'}-\left( -1 \right)^{j-1}\boldsymbol{c}_{j}^{2} \right| \right)e^{\left( -1 \right)^{j}i\boldsymbol{k}\boldsymbol{c}_{\boldsymbol{j}}^{\boldsymbol{2}}} & 0 \end{matrix} \right),$(16)

$F=-\left( \begin{matrix} t\left( \left| \boldsymbol{P}_{a_{1}}^{'}-\boldsymbol{P}_{a_{2}}^{'} \right| \right) & t\left( \left| \boldsymbol{P}_{a_{1}}^{'}-\boldsymbol{P}_{b_{2}}^{'} \right| \right) \\ t\left( \left| \boldsymbol{P}_{a_{2}}^{'}-\boldsymbol{P}_{b_{1}}^{'} \right| \right) & t\left( \left| \boldsymbol{P}_{b_{1}}^{'}-\boldsymbol{P}_{b_{2}}^{'} \right| \right) \end{matrix} \right),$ (17)

where $\boldsymbol{c}_{1}^{1(2)}$ and $\boldsymbol{c}_{2}^{1(2)}$ represent the basis vectors $(\frac{a}{2},\frac{\sqrt{3}a}{2},0)$ and $(\frac{a}{2},-\frac{\sqrt{3}a}{2},0)$ for layer 1 or 2 respectively. $\boldsymbol{k}$ is the wavevector $\left( k_{x},k_{y},0 \right)$. For the twist angle $\theta\in\left[ 0^{^{\circ}},{60}^{^{\circ}} \right]$, $t_{1}=t\left( \left| \boldsymbol{P}_{b_{1}}^{'}-\boldsymbol{P}_{a_{1}}^{'}+\boldsymbol{c}_{1}^{1} \right| \right)=t\left( \left| \boldsymbol{P}_{a_{1}}^{'}-\boldsymbol{P}_{b_{1}}^{'}-\boldsymbol{c}_{1}^{1} \right| \right)=t\left( \left| \boldsymbol{P}_{b_{2}}^{'}-\boldsymbol{P}_{a_{2}}^{'}-\boldsymbol{c}_{2}^{2} \right| \right)=t\left( \left| \boldsymbol{P}_{a_{2}}^{'}-\boldsymbol{P}_{b_{2}}^{'}+\boldsymbol{c}_{2}^{2} \right| \right)=A_{0}e^{-\gamma\sqrt{\frac{2}{3}[2+\sqrt{3}sin(\frac{\theta}{2}-\frac{\pi}{3})]}}$, $t_{2}=t\left( \left| \boldsymbol{P}_{b_{1}}^{'}-\boldsymbol{P}_{a_{1}}^{'}-\boldsymbol{c}_{2}^{1} \right| \right)=t\left( \left| \boldsymbol{P}_{a_{1}}^{'}-\boldsymbol{P}_{b_{1}}^{'}+\boldsymbol{c}_{2}^{1} \right| \right)=t\left( \left| \boldsymbol{P}_{b_{2}}^{'}-\boldsymbol{P}_{a_{2}}^{'}+\boldsymbol{c}_{1}^{2} \right| \right)=t\left( \left| \boldsymbol{P}_{a_{2}}^{'}-\boldsymbol{P}_{b_{2}}^{'}-\boldsymbol{c}_{1}^{2} \right| \right)=A_{0}e^{-\gamma\sqrt{\frac{2}{3}\left[ 2-\sqrt{3}\sin\left( \frac{\theta}{2}+\frac{\pi}{3} \right) \right]}}$, as shown in Fig. S2. The terms in $F$ are $f_{11}=-t\left( \left| \boldsymbol{P}_{a_{1}}^{'}-\boldsymbol{P}_{a_{2}}^{'} \right| \right)=-A_{0}e^{-\gamma\sqrt{h^{2}+\left( 4r_{0}^{2}+\frac{a^{2}}{3} \right){sin}^{2}\frac{\theta}{2}}}$, $f_{22}=-t\left( \left| \boldsymbol{P}_{b_{1}}^{'}-\boldsymbol{P}_{b_{2}}^{'} \right| \right)=-A_{0}e^{-\gamma\sqrt{h^{2}+\left( 4r_{0}^{2}+\frac{a^{2}}{3} \right){sin}^{2}\frac{\theta}{2}}}$, $f_{12}=-t\left( \left| \boldsymbol{P}_{a_{1}}^{'}-\boldsymbol{P}_{b_{2}}^{'} \right| \right)=-A_{0}e^{-\gamma\sqrt{h^{2}+\frac{a^{2}}{3}+\left( 4r_{0}^{2}-\frac{a^{2}}{3} \right){sin}^{2}\frac{\theta}{2}-\frac{2}{\sqrt{3}}ar_{0}sin\theta}}$ and $f_{21}=-t\left( \left| \boldsymbol{P}_{a_{2}}^{'}-\boldsymbol{P}_{b_{1}}^{'} \right| \right)=-A_{0}e^{-\gamma\sqrt{h^{2}+\frac{a^{2}}{3}+\left( 4r_{0}^{2}-\frac{a^{2}}{3} \right){sin}^{2}\frac{\theta}{2}+\frac{2}{\sqrt{3}}ar_{0}sin\theta}}$. Here, the critical nature of evolution from AA to AB/BA is concentrated in six azimuths $\theta_{c}$. $|f_{11}|$ and $|f_{22}|$ are equal and experience simultaneously a continuous decrease as $r_{o}$ increases, i.e., $|f_{11}|,|f_{22}|$ are negatively correlated to $r_{o}$. For the certain $\theta_{c}$, e.g., 0, the $|f_{12}|$ shows a significant increase as $r_{o}$ increases for $r_{o}\in(0,\frac{a}{2sin\theta/2})$ (which is the most important range since it just satisfies an evolution from AA to AB/BA, i.e., the lattice constant of natural superlattice is approximate to $\frac{a}{2sin\theta/2}$) and $|f_{21}|\to0$. While for the opposite direction (i.e., $\pi$), $|f_{21}|$ shows a consistent trend and |$f_{12}|\to0$. At this time, due to the 180^o^ rotation, $|f_{12}|$ and $|f_{21}|$ have exchanged.


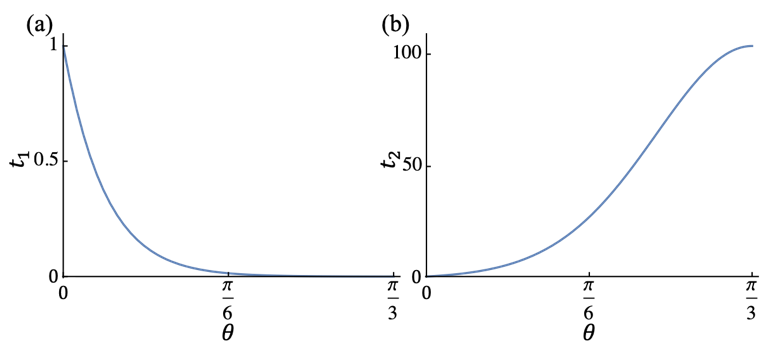


FIG. S2. (a) $t_{1}$ and (b) $t_{2}$ varied with the twist angle $\theta$.

**Supplementary Note 3: The minimal two-level model for paired superflat bands**

Superflat band phenomenology in our system can be understood as interacted localized states in periodic moiré superlattices with tiny inter-cell coupling. Localized states with $C_{3}$ and $C_{6}$ symmetries, i.e., $\Phi_{TLM}=\{\phi_{C_{3}},\phi_{C_{6}}\}$, for periodic systems, yields the minimal two-level model (TLM) Hamiltonian

$H_{TLM}=\left( \begin{matrix} -m & \kappa_{1}+\sum_{i=1}^{6} \kappa_{2}e^{-\boldsymbol{a}_{M}^{i}\cdot\boldsymbol{k}} \\ \kappa_{1}+\sum_{i=1}^{6} \kappa_{2}e^{\boldsymbol{a}_{M}^{i}\cdot\boldsymbol{k}} & m \end{matrix} \right),$ (18)

where $m$ acts as the mass terms, $\kappa_{1}$ and $\kappa_{2}$ denote inter-cell coupling coefficients between two localized states, respectively. $\boldsymbol{a}_{M}^{i}=\left| \boldsymbol{a}_{M}^{i} \right|\{\cos\left( \frac{2i-1}{6}\pi\right),sin(\frac{2i-1}{6}\pi)\}$ represent moiré superlattice basis vectors for all six directions with $\left| \boldsymbol{a}_{M}^{i} \right|=\frac{a}{2\sin\left( \frac{\theta}{2} \right)}$ and $\boldsymbol{k=}\{k_{x},k_{y}\}$, $i=0,1,\ldots,5$. $H_{TLM}$ is constrained by the conditions $\kappa_{2}\ll\kappa_{1},m$, and implies massive Dirac physics. Specifically, stable and tiny $\kappa_{2}$ indicates a strong spatial confinement in the AA stacked region, trending to realize near-zero dispersion. By fitting band structures calculated by the TBM, we extract $m$ and $\kappa_{1,2}$ as functions of $\theta$. As illustrated in Fig. S3 (a), $\kappa_{1,2}$ grow as $\theta$ increases indicating that localized state diffused at large $\theta$ promotes the overlap between them instead (despite the significant impact for $\kappa_{1}$ and subtle impact for $\kappa_{2}$), while $m$ reduces as $\theta$ increases until it merges to bulk energies of AA/BA stacked lattices.

According to Eq. (18), the flatness of superflat bands can be well defined using the energy difference between high symmetry points $K_{S}$ and $\Gamma_{S}$ in moiré BZs, and reads

$\Delta_{g}=\frac{{9\kappa}_{2}}{\sqrt{\left( \frac{m}{\kappa_{1}} \right)^{2}+1}}\mathcal{+O}\left( \kappa_{2}^{2} \right).$ (19)

Ideal minimal TLM Hamiltonian of Eq. (18) has chiral symmetry, i.e., $\sigma_{z}H_{TLM}\sigma_{z}=-H_{TLM}$, and guarantees that two isolated band structures $\Xi_{-}$ and $\Xi_{+}$ share the same $\Delta_{g}$. Here only one parameter $\kappa_{2}\to0$ enforces the perfect superflat bands $\Delta_{g}\to0$. In Fig. S3 (a), minimal TLM results indicate that the ideal superflat bands always appear in a wide and continuous range of $\theta$. We also display the theoretical $\Delta_{g}$ changed with $\theta$ based upon three parameters in Fig. S3 (a). Compared with minimal TLM results, TBM calculation presents that the flatness of $\Xi_{-}$ and $\Xi_{+}$ splits because of the perturbation of a considerable number of bulk bands (for both AA and AB/BA stacked lattices). Owing to a small $\theta$ endowing a large $m$, isolated $\Xi_{-}$ and $\Xi_{+}$ rarely cause chiral symmetry to be broken. Slightly small $\theta$ allows for perfect superflat bands, for example the case of $\theta\leq{6.01}^{\circ}$, see Fig. S3 (b). As shown in Fig. S3 (c), accurate TBM calculation gives the typical eigenstates at $\Gamma_{S}$ point of $\Xi_{-}$ and $\Xi_{+}$ for $\theta={6.01}^{\circ}$.


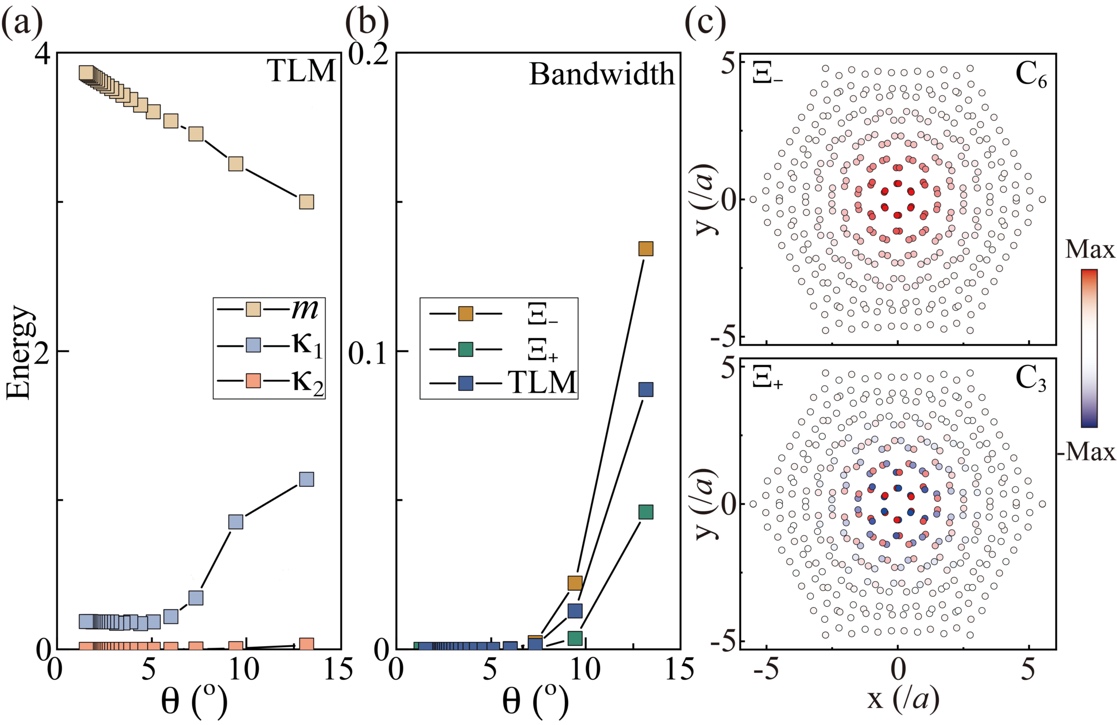


FIG. S3. (a) Minimal TLM parameters $m$ and $\kappa_{1,2}$ fitted according to the TBM, with $h=a/\sqrt{3}$. As $\theta$ increases, $m$ tends to the bulk energies of AB/BA stacked lattices. $\kappa_{1}$ increases as $\theta$ increases indicating the enhanced intracell overlap between two localized states. $\kappa_{2}$ stays close to zero in a wide range of $\theta$. (b) Bandwidth $\Delta_{g}$ calculated by minimal TLM and TBM for $\Xi_{-}$ and $\Xi_{+}$. For the highest and lowest bands, $\Delta_{g}$ are the same for minimal TLM due to the chiral symmetry while TBM calculation results present limited symmetry breaking. For small $\theta$, $\Delta_{g}\to0$ always holds, for example, $\theta\leq{6.01}^{\circ}$. (c) Typical eigenstates located at the highest and lowest energies of $\Gamma_{S}$ point for moiré BZs, exhibiting an obvious localized character with $C_{3}$ and $C_{6}$ symmetries, respectively.

**Supplementary Note 4: Nanophotonic implementation in the open system**

The exotic phenomena in the main text can theoretically exist in open systems. To demonstrate this, we simulate an open system containing silicon triangular prisms (refractive index 3.46) and a polymethyl methacrylate (PMMA) background (refractive index 1.47). Single PC slab has a $C_{6v}$ lattice with lattice constant $a_{Si}=1.5um$. The triangular prisms have the side length $0.5a_{Si}$ and height $0.4a_{Si}$. The PMMA layer between two PC slabs has a thickness of $0.2a_{Si}$. The whole structure is placed in PMMA environment with open boundaries along the z-axis.

First, for ideal AA and AB/BA stacked twisted bilayer PC slabs, we give the band structures for $C_{3}$ symmetric modes near $\Gamma$ point as shown in Figs. S4 (a) and (b). The potential difference for the highest frequencies can be clearly observed, which implies the presence of potential wells. We further provide the spectrum of twisted bilayer PC slabs near $\Gamma_{S}$ point. An isolated frequency appears in the gap of bulk spectrum corresponding to the localized state with $C_{3}$ symmetry as displayed in Figs. S4 (c) and (d). The localized states can exist stably in open systems, exhibiting well wave confinement capabilities.


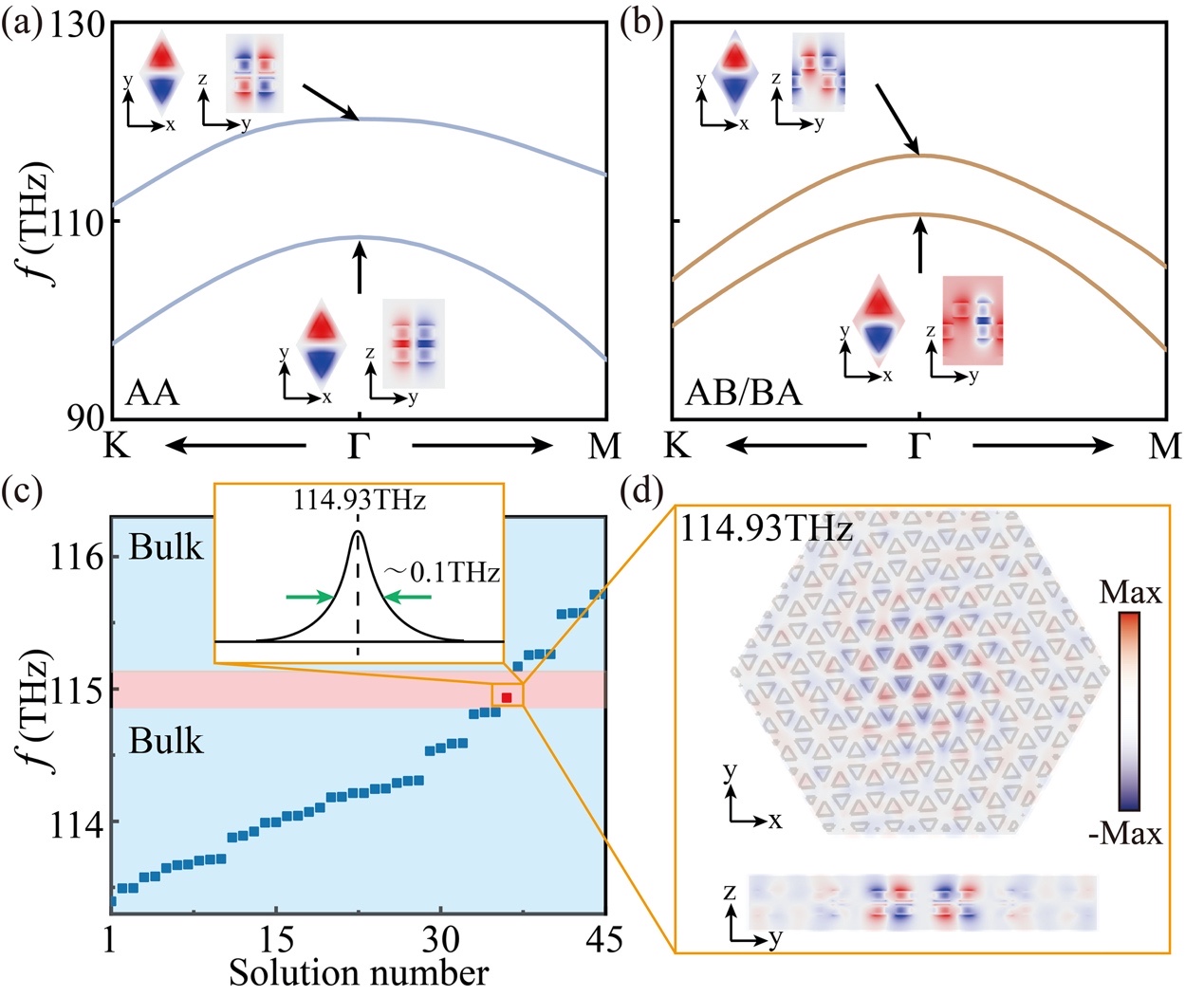


FIG. S4. (a) Band structures of AA stacked PCs near $\Gamma$ point for upper two bands. (b) Band structures of AB/BA stacked PCs near $\Gamma$ point for upper two bands. (c) Spectrum near $\Gamma_{S}$ point for twisted bilayer PC slabs with isolated frequency 114.93THz. (d) Field distribution of localized states at 114.93THz.

**Supplementary Note 5: The absence of localized states with** $\boldsymbol{C}_{\boldsymbol{6}}$ **symmetry in lowest bands of all-dielectric photonic crystals**

In the low-frequency limit, the all-dielectric PC behaves like a homogeneous medium^3^. As shown in Fig. S5, both AA and AB/BA stacked PC slabs have a linear dispersion relation in the lowest band near $\Gamma$ point. According to our TBM for AA and AB/BA lattices, these bands correspond to $C_{6}$ symmetries, which are the origin of the generation of localized states with $C_{6}$ symmetric lattice fields. However, they are bound to “zero” frequency of $\Gamma$ point, giving rise to the absence of potential well for entire moiré superlattices. The chiral symmetry is broken, and the band structure is beyond the scope of the TBM with nearest-neighbor couplings. Hence the localized states with $C_{6}$ symmetric lattice fields must disappear and lead to the absence of superflat bands for the $C_{6}$ symmetry.


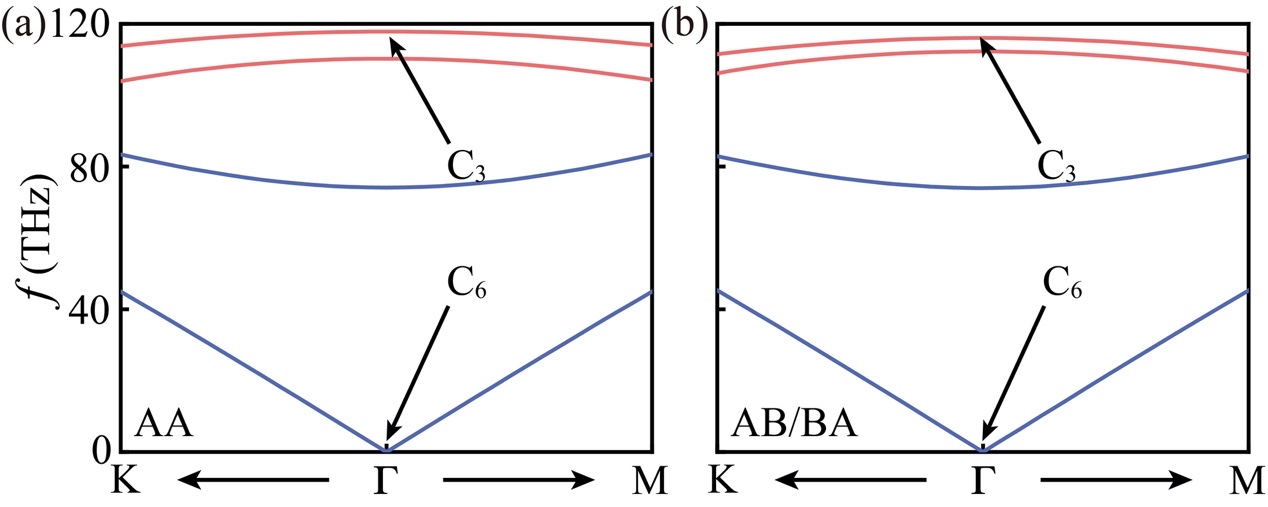


FIG. S5. (a) Band structures of AA stacked PC slabs in Fig. 4 near $\Gamma$ point for lowest four bands. (b) Band structures of AB/BA stacked PC slabs in Fig. 4 near $\Gamma$ point for lowest four bands.

**Supplementary Note 6: The robustness of superflat bands**

Superflat bands in our system appear in a wide range of parameters. This depends on the conditions of existence of potential well, which are broken only when the interlayer distance tends to infinity. The increase in twist angles also disrupts the degree between AA and AB/BA stacked lattice evolution, thus causing a gradual change in superflat bands. As shown in Fig. S6, we use the TBM showing that the superflat bands are clearly present until the twist angle ${13.17}^{\circ}$, and then gradually merges into the bulk bands as twist angles increase. Therefore there is no critical twist angle limit for the appearance of superflat bands in our system. Since this is a collective effect caused by lattice distortion protected by the superlattice symmetry, non-extreme parameters are always able to maintain the appearance of such superflat bands and localized states.

Here, we have chosen the most general cylinder to show the robustness of superflat bands and corresponding localized states in the nanophotonic platform. Unit cells for single PC slabs have the lattice constant $a_{Si}=1.5um$. The cylinders are selected as silicon materials with radius $0.15a_{Si}$ and height $0.4a_{Si}$ (the background is air). The gap between two PC slabs is $0.2a_{Si}$. The entire structure has perfect metal boundary conditions in the z-direction, similar to Fig. 4. As shown in Fig. S7 (a), the superflat bands remain stable even if they are located in the frequency range of the bulk spectrum. This is because the superflat bands always have the states with extremely strong confinement capabilities, which are orthogonal to higher-order states even if the band crossing happens, as displayed in Fig. S7 (b). In this sense, the general system parameter does not affect the existence of superflat bands and corresponding localized states.


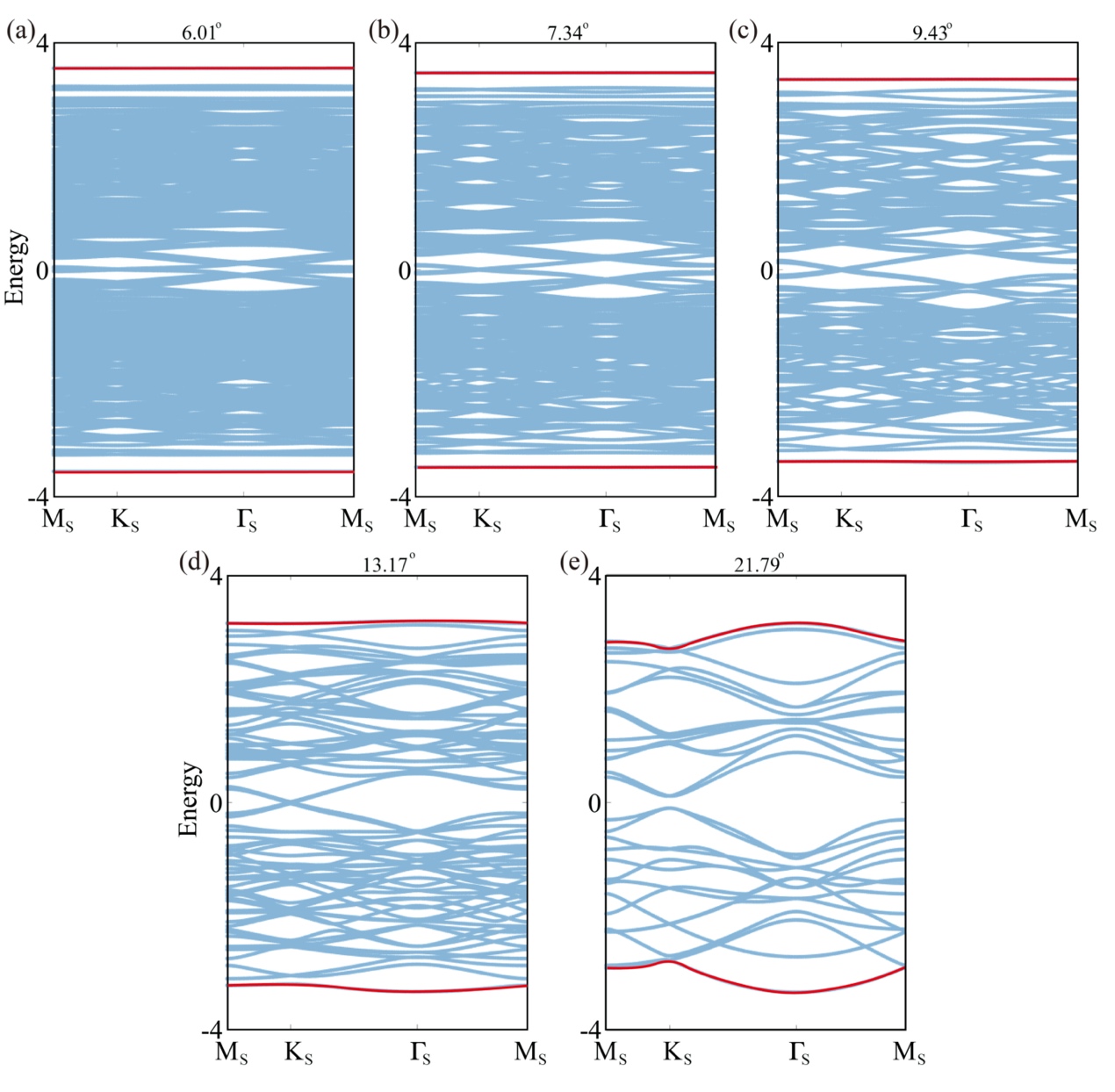


FIG. S6. Band structures of twist bilayer systems with different twist angles calculated by the TBM with $h=a/\sqrt{3}$. (a)-(e) correspond to ${6.01}^{\circ}$, ${7.34}^{\circ}$, ${9.43}^{\circ}$, ${13.17}^{\circ}$ and ${21.79}^{\circ}$, respectively. Red lines label the corresponding superflat bands while blue lines represent bulk bands.


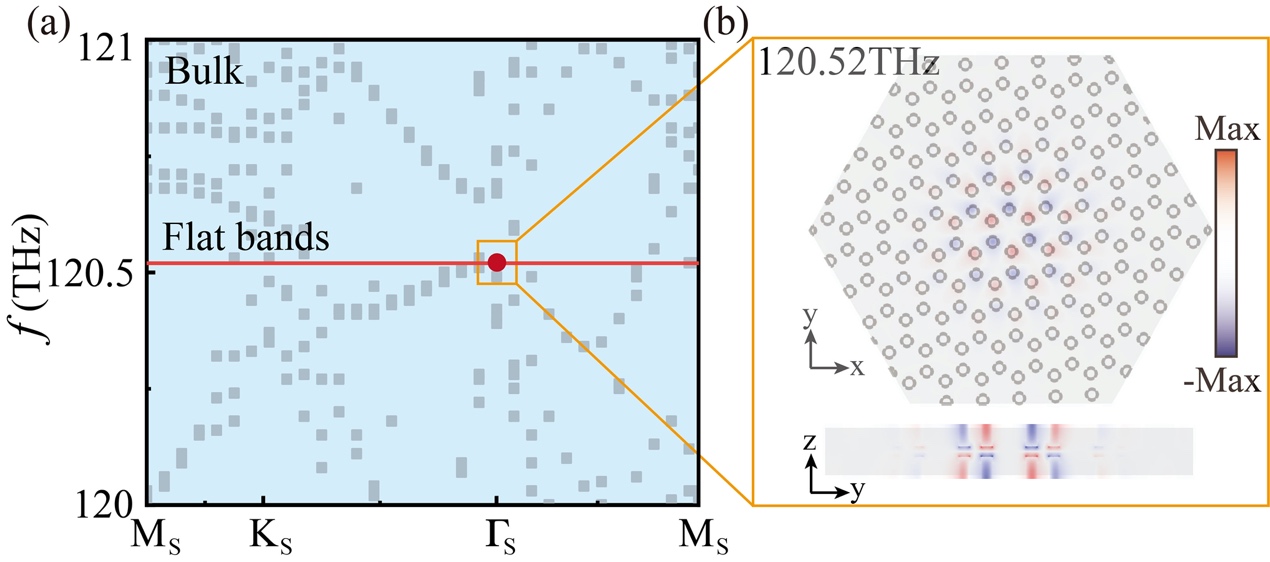


FIG. S7. (a) Band structures of twist bilayer PC slabs with the twist angle ${6.01}^{\circ}$, where the red line (gray dot) represents the superflat (bulk) band. (b) The localized state at the $\Gamma_{S}$ point of superflat bands.

**Supplementary Note 7: The impact of nonzero** $\boldsymbol{|\epsilon|}$ **for superflat bands**

In our analysis process, the nonzero $|\epsilon|$ directly leads to the correction of bulk energies of AA AB/BA stacked lattices. For AA stacked lattices, the highest and lowest energies can be corrected as

${max: E}_{\Gamma}^{AA}=\sqrt{{t_{ij}\left( h \right)}^{2}+\epsilon^{2}}+3t_{0}$, (20)

${min: E}_{\Gamma}^{AA}=-(\sqrt{{t_{ij}\left( h \right)}^{2}+\epsilon^{2}}+3t_{0})$. (21)

For AB/BA stacked lattices, the highest and lowest energies can be corrected as

${max: E}_{\Gamma}^{AB/BA}=\sqrt{{t_{ij}\left( h \right)}^{2}+2\epsilon^{2}+18t_{0}^{2}+\sqrt{{t_{ij}\left( h \right)}^{4}+36{t_{ij}\left( h \right)}^{2}t_{0}^{2}+144\epsilon^{2}t_{0}^{2}}}$, (22)

${min: E}_{\Gamma}^{AB/BA}=-\sqrt{{t_{ij}\left( h \right)}^{2}+2\epsilon^{2}+18t_{0}^{2}+\sqrt{{t_{ij}\left( h \right)}^{4}+36{t_{ij}\left( h \right)}^{2}t_{0}^{2}+144\epsilon^{2}t_{0}^{2}}}$. (23)

According to above equations, we notice that nonzero $\left| \epsilon\right|$ causes the energy difference to shift to the outside. This also results in the energy shift of localized states, which further causes the absolute energies of superflat bands to move to large value. Such a process is equivalent to introducing a correction to $m$ of the minimal TLM

$m\to m+g\left( \left| \epsilon\right| \right),$ (24)

where $g\left( \left| \epsilon\right| \right)$ can be extracted by fitting the TB calculation. We further calculate the energies of superflat bands changed with $\left| \epsilon\right|$ using the TB model as shown in Fig. S8 (a). A specific case is displayed in Fig. S8 (b) showing the exact band structures with $\left| \epsilon\right|=0.5$. Superflat bands appear in isolation at the highest and lowest positions stably.


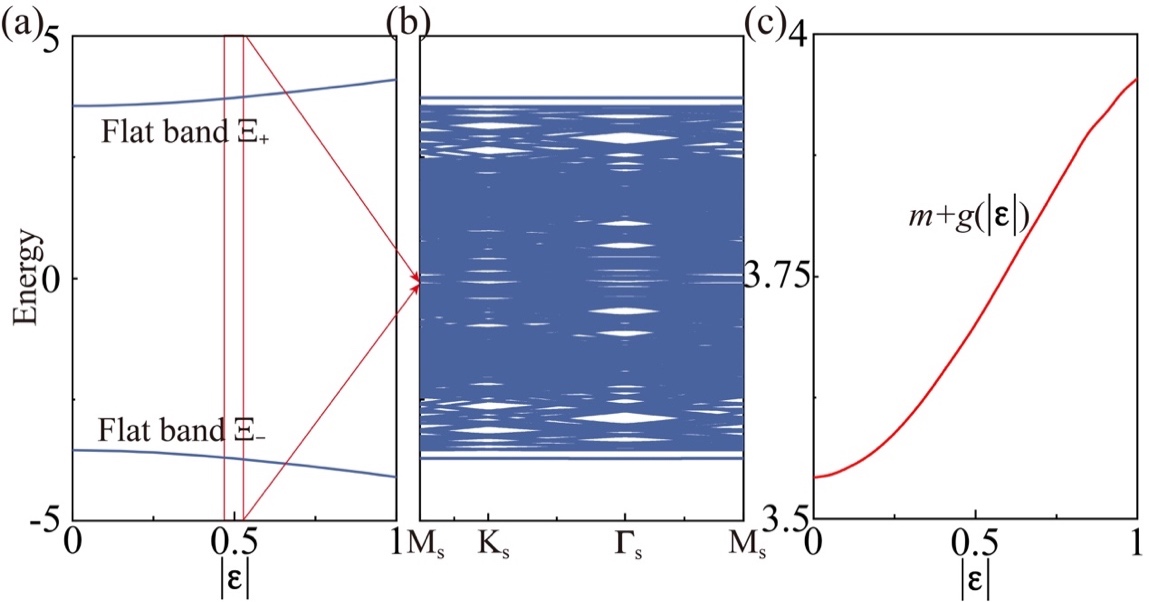


FIG. S8. (a) The energies of superflat bands varied with $\left| \epsilon\right|$. The absolute energies grow as $\left| \epsilon\right|$ increases with $h=a/\sqrt{3}$ and $\theta={6.01}^{o}$. (b) Band structures of twisted bilayer systems with $\left| \epsilon\right|=0.5$. (c) Extracted $m+g\left( \left| \epsilon\right| \right)$ fitted by TBM calculations.

We use the minimal TLM to fit the parameters as shown in Fig. S8 (c). Results show that $g\left( \left| \epsilon\right| \right)$ is always greater than zero, which can also be derived from Eqs. (20)-(23) (related to $\epsilon^{2}$), i.e., $\propto\left| \epsilon\right|$. So that, $g\left( \left| \epsilon\right| \right)$ grows as $\left| \epsilon\right|$ increases. In conclusion, nonzero $\left| \epsilon\right|$ does not determine the presence of superflat bands but shifts their absolute energies to the higher level.

**References**

[1] Fang C., Weng H., Dai X. & Fang Z. Topological nodal line semimetals. Chin. Phys. B **25**, 117106 (2016).

[2] Montambaux G., Lim L.-K., Fuchs J.-N. & Piéchon F. Winding Vector: How to Annihilate Two Dirac Points with the Same Charge. Phys. Rev. Lett. **121**, 256402 (2018).

[3] Krokhin A. A., Halevi P. & Arriaga J. Long-wavelength limit (homogenization) for two-dimensional photonic crystals. Phys. Rev. B **65**, 115208 (2002).
